# Errors on the Trail Making Test Are Associated with Right Hemispheric Frontal Lobe Damage in Stroke Patients

**DOI:** 10.1155/2015/309235

**Published:** 2015-05-13

**Authors:** Bruno Kopp, Nina Rösser, Sandra Tabeling, Hans Jörg Stürenburg, Bianca de Haan, Hans-Otto Karnath, Karl Wessel

**Affiliations:** ^1^Cognitive Neurology, Technische Universität Braunschweig and Department of Neurology, Braunschweig Hospital, Salzdahlumer Street 90, 38126 Braunschweig, Germany; ^2^Department of Neurology, Hannover Medical School, Carl-Neuberg Street 1, 30625 Hannover, Germany; ^3^Klinik Niedersachsen, Haupt Street 59, 31542 Bad Nenndorf, Germany; ^4^Division of Neuropsychology, Center of Neurology, Hertie Institute for Clinical Brain Research, University of Tübingen, Hoppe-Seyler Street 3, 72076 Tübingen, Germany; ^5^Department of Psychology, University of South Carolina, 1512 Pendleton Street, Columbia, SC 29208, USA

## Abstract

Measures of performance on the *Trail Making Test (TMT)* are among the most popular neuropsychological assessment techniques. Completion time on *TMT-A* is considered to provide a measure of processing speed, whereas completion time on *TMT-B* is considered to constitute a behavioral measure of the ability to shift between cognitive sets (cognitive flexibility), commonly attributed to the frontal lobes. However, empirical evidence linking performance on the *TMT-B* to localized frontal lesions is mostly lacking. Here, we examined the association of frontal lesions following stroke with *TMT-B* performance measures (i.e., completion time and completion accuracy measures) using voxel-based lesion-behavior mapping, with a focus on right hemispheric frontal lobe lesions. Our results suggest that the number of errors, but not completion time on the *TMT-B*, is associated with right hemispheric frontal lesions. This finding contradicts common clinical practice—the use of completion time on the *TMT-B* to measure cognitive flexibility, and it underscores the need for additional research on the association between cognitive flexibility and the frontal lobes. Further work in a larger sample, including left frontal lobe damage and with more power to detect effects of right posterior brain injury, is necessary to determine whether our observation is specific for right frontal lesions.

## 1. Introduction

Trail making tasks are popular neuropsychological tests [1, 2], because of their ease of administration and the presumed utility as sensitive measures of brain dysfunction [3]. The most widespread trail making task is* Trail Making Test, Parts A and B* (*TMT*; [4]). On* TMT-A*, one connects 25 encircled numbers randomly arranged on a page in ascending order by drawing a pencil line (i.e., 1-2-3⋯25). It is the same on* TMT-B*, except that 25 encircled numbers and letters need to be connected in alternating order (i.e., 1-A-2⋯12-L-13).

Trail making tasks were originally based on a test measuring divided attention,* Partington's Pathways *[5]. Halstead [6] recognized its potential for his studies of the biological basis for intelligence. Many other trail making tasks are available meanwhile (e.g., [7]; see [3], for review). Perceptual/motor speed, speed of cognitive processing, (divided) attention, visual search, working memory, executive control, cognitive flexibility, and general intelligence contribute to* TMT* performance. However, there is no consensus about their exact nature and relative contributions (see [8] for review). There exists a substantial correlation between* TMT-A* and* TMT-B* completion times [9], suggesting that they measure “… similar although somewhat different functions” [3, page 668]. Executive abilities (see [10], for discussion) play an important role in* TMT-B* performance. According to Kortte et al. [11],* TMT-B* performance correlates more closely with cognitive flexibility measures (i.e., perseveration errors on the* Wisconsin Card Sorting Test*) (*WCST* [12]; see [13], for review) than with working memory measures (i.e., failure to maintain set score on* WCST*). The consistent finding of substantial correlations between* TMT-B* and* WCST* perseverative indices suggests cognitive flexibility being among the key executive abilities underlying performance on the* TMT-B* (e.g., [11]).

*TMT* completion time measures are sensitive to the presence of various neurological and psychiatric disorders [14], but their diagnostic utility in* differential* diagnosis has repeatedly been questioned [15–17]. The generally accepted linkage between cognitive flexibility and frontal lobe functioning (e.g., [18]) suggests that the* TMT-B* could be used to evaluate frontal lobe (dys)function. Ricker et al. [19] found* TMT-B* completion time being related to frontal lobe dysfunction. However, this finding must be treated cautiously given negative results in studies comparing patients with frontal and posterior brain damage on* TMT*-*B* completion time [20, 21]. A meta-analysis by Demakis [22] found significant group differences only for completion time on* TMT-A,* but the effect size was small, indicating little separation between frontal and posterior groups and relatively poor* TMT-A* sensitivity and specificity. Bonilha et al. [23] investigated the relationship between prefrontal cortical atrophy and neuropsychological performance in schizophrenic patients and found that decreased Brodmann's area (BA9) grey matter volume correlated with poorer task performance on* WCST* errors and* TMT-B* completion time.

Taken together, the association between* TMT-B* completion time measures and frontal lobe dysfunction seems to be relatively weak or even absent. Against this background, it is often thought that prolongation of* TMT-B* completion time in the presence of normal* TMT-A* completion time does suggest frontal dysfunction. Specifically, subtracting* TMT-A* from* TMT-B* completion time is a common method for partialling out effects of general processing speed difficulties that patients might have [1]. Although this subtraction method is widely used in clinical practice, we deliberately decided to refrain from examining whether* TMT-B* minus* TMT-A* completion time data correlate with relevant brain regions. Our reluctance can be traced back to the fact that the difference between two substantially correlated measures, as in the case of* TMT-A* and* TMT-B* completion times, possesses unacceptable low levels of reliability, thereby precluding its potential use in clinical practice (see [9], for the rationale behind this recommendation).

Thus, there remains a need for additional research on potential associations between cognitive flexibility, as assessed by the* TMT*, and frontal lobes.* TMT-B* completion accuracy represents a promising candidate measure in that regard since a former study suggested a relationship between* TMT-B* completion accuracy measures and frontal brain dysfunctions [24]. Analysis of errors on* TMT-B* in Stuss et al.'s [24] study indicated that all patients who made two or more than two errors had frontal lesions (but see [17], for a failure to replicate Stuss et al.'s [24] finding). Further, dividing the frontal damaged patients into subgroups on the basis of the number of errors yielded specificity of brain-behavior relations within the frontal lobes: patients with damage in dorsolateral frontal areas were most impaired, while those with damage to the medial frontal lobes were not significantly affected on* TMT-B* completion accuracy.

Klusman et al. [25] distinguished between* TMT-B shifting* (e.g., connecting 1-2⋯ or A-B⋯) and* sequencing* errors (e.g., connecting 1-A-3⋯ or A-2-C⋯). Stuss et al. [24] found notable slowing of* TMT-B* in patients with frontal lobe damage, but they concluded error analysis providing a more useful method to differentiate between frontal and posterior brain damage because all patients committing two or more* TMT-B* errors had frontal lesions. Notably, many patients with lesions in the dorsolateral prefrontal cortex (DLPFC) committed two or more errors (irrespective of error type), whereas ventrolateral prefrontal and orbitofrontal lesions did not affect* TMT-B* accuracy variables comparably. Stuss and Levine [18, page 415] concluded the following: “*TMT-B* errors (but not time), therefore, are a valid measure of DLPFC dysfunction.”

In the present study, we investigate the association between* TMT* completion time and accuracy measures with frontal lobe damage in stroke patients using voxel-based lesion-behavior mapping [26–28]. In contrast to traditional overlap designs [26] in which the overlapping of lesion boundaries in individual patients from different groups limits study validity (cf. [26]), voxel-based lesion-behavior analysis yields a statistical approach to uncover brain-behavior relationships without any prior patient categorization. Moreover, previous research on behavioral effects of frontal brain damage often compared patient groups with heterogeneous etiological lesions (see [29]). These shortcomings notwithstanding and based on Stuss et al. [24], we hypothesize that* TMT-B* completion accuracy measures, but not* TMT-B* completion time measures, are sensitive to DLPFC damage.

## 2. Method

### 2.1. Subjects

Thirty acute, first-ever, and right-hemisphere-damaged stroke patients with damage involving the frontal lobe in most patients participated in the study (see Table 1). These neuropsychological results show that our sample consisted of stroke patients without generalized cognitive deficits as revealed by the Mini-Mental-State-Examination (*MMSE*, [32]) and without notable verbal disturbances as revealed by the Wortschatz-Test (Vocabulary Test) (*WST*, [35]) and by the Regensburger Wortflüssigkeits-Test (Regensburger Word Fluency Test) (*RWT*, [34]). Further, the Modified Card Sorting Test (*MCST*, [33]) (a variant of the* WCST*) data suggest that cognitive flexibility and working memory were not severely disturbed in our patients.

The logic in restricting to right hemispheric strokes was to exclude patients with paresis of the dominant right hand and/or apraxia, possibly distorting task performance. Further, left-hemisphere strokes might have hampered the understanding of task instructions, due to potential presence of sensory aphasia. 1 Patients with traumatic brain injury, brain tumours, subcortical arteriosclerotic encephalopathy, neurodegenerative disease, or gross neurological defects (pronounced pain reported by the patient, left homonymous hemianopia revealed by clinical examination, and hemispatial visual neglect) were excluded to ensure symptoms did not interfere with task performance. 1 Spatial neglect was diagnosed when a patient oriented toward the ipsilesional side when addressed from the front or left and/or ignored contralesionally located people or objects. Patients without prior psychiatric disease, alcohol, or drug abuse were recruited.

In many studies, patients with lesions from many different etiologies are assessed (e.g., patients with traumatic brain injury and brain tumors). Here, we restricted ourselves to acute stroke patients (most often due to infarcts of the middle cerebral artery, a minority with anterior or posterior cerebral artery infarcts or with hemorrhagic stroke) in order to assure that the full neurological damage did not extend considerably beyond the borders of the visible lesion. As a consequence, the cognitive deficits displayed by the majority of our patients are most likely (solely) related to the visible lesion.

All patients gave their informed written consent to participate in the study, in accordance with the ethical standards of the Declaration of Helsinki (1964). Appropriate ethical approval for the study was obtained from the Ethics Committee at Technische Universität Braunschweig (Faculty for Life Sciences; ref. 37-2010).

### 2.2. Materials

#### 2.2.1. Test Description

Each patient performed the* TMT-A* and* TMT-B* as described above. Before each test trial, a practice trial of six items was administered to ensure task understanding. Participants were instructed to perform the test as fast and as accurately as possible. During performance, each error was immediately corrected by the examiner instructing the participant to go back with his pencil to the last correct item. All participants continued until they completed the task. Raw completion times and number of errors were the dependent measurements.

One error type on* TMT-A* (i.e., one type of sequencing error) and four error types on* TMT-B* (i.e., two types of sequencing and shifting errors, resp.) were classified according to Klusman et al. [25]. In making a shifting error, participants failed to alternate correctly either from a number to a letter (Type A error; e.g., 1-A-2-3; incorrect choices are underlined) or from a letter to a number (Type B error; e.g., 1-A-B). A sequencing error occurred when participants incorrectly sequenced either numbers (Type C error; e.g., 1-3 on* TMT-A* or 1-A-3 on* TMT-B*) or letters (Type D error; e.g., 1-A-2-C on* TMT-B*).

### 2.3. Lesion Analysis

Magnetic resonance imaging (MRI) was performed in 27 stroke patients and computed tomography (spiral CT) scanning in three stroke patients. The initial scanning was repeated until the infarcted area became clearly demarcated. The mean time interval between lesion onset and MRI scan amounted to 3.9 days (SD = 3.2), between lesion onset and CT scanning to 2.6 days (SD = 3.7). MRI scans were obtained on a 1.5 T echo planar imaging (EPI) capable system (Philips Intera, Philips Medical Systems, Best, Netherlands). The MRI protocol used diffusion-weighted imaging (DWI, *N* = 11) and T_2_-weighted fluid-attenuated inversion-recovery imaging (FLAIR, *N* = 16). DWI was performed with a single-shot EPI spin echo sequence (25 axial slices; repetition times (TR), either 3690, 4000, 4452, 5060, 5300, or 6360 ms; echo times (TE), either 90, 95, or 120 ms; field of view (FOV), 230 × 230 mm^2^; matrix 64 × 64 pixels; slice thickness, 5 mm; gap, 5.5 mm). FLAIR sequences were acquired with 25 axial slices (thickness, 5 mm) with an interslice gap of 5.5 mm, FOV of 220 × 220 mm^2^, TR of either 4000, 5397, 5500, or 6000 ms, and TE of either 89, 91, 100, or 120 ms. CTs were obtained on a spiral scanning system (Somatom Sensation 16, Siemens Healthcare, Erlangen, Germany) with a slice thickness of 3 mm infratentorial and 6 mm supratentorial (and an in-plane resolution of 0.5 × 0.5 mm).

Lesion location was evaluated using* MRIcroN* ([27]; http://www.mricro.com/). In MRI scans, lesion boundaries were delineated directly on the individual scans. MRI scan and lesion shape were then mapped into stereotaxic space using the normalization algorithm in SPM5 (http://www.fil.ion.ucl.ac.uk/spm/). Cost-function masking was employed for determination of transformation parameters [36].

In spiral CT scans, lesions were drawn directly by an experienced neurologist (H.-O. K., blinded for test performance) on the slices of a normalized T_1_-weighted template MRI scan from the Montreal Neurological Institute (MNI) with a 1 × 1 mm in-plane resolution, distributed with* MRIcroN*. Lesions were mapped onto the slices that correspond to MNI *Z*-coordinates (−16, −8, 0, 8, 16, 24, 32, and 40 mm) by using the identical or closest matching axial slices of each individual patient.

To evaluate the relationship between lesion location and performance on* TMT-A* and* TMT-B*, a voxel-based lesion-behavior analysis was performed using the Brunner-Munzel (BM) test [37] for continuous variables implemented in* MRIcroN* ([27]; http://www.mricro.com/). Only voxels (*N* = 146.224) damaged in at least three patients were included in the analysis. We controlled for multiple comparisons applying permutation-based thresholding [38] using 4000 iterations. All results presented survived a 5% permutation-based false positive probability threshold.

## 3. Results

### 3.1. Neuropsychological Results

Tables 2 and 3 summarize patients'* TMT* performance. Average completion times showed the typical prolongation of* TMT-B* compared to* TMT-A*. Sequencing errors on* TMT-A* occurred relatively infrequently (6.7% of patients committed ≥ 2 errors). Total errors on* TMT-B* were much more common (43% of patients committed ≥ 2 errors). Type B (13.3% of patients committed ≥ 2 errors) outnumbered Type A (6.7% of patients committed ≥ 2 errors) shifting errors, indicating that the failure to shift from a letter to a number was more prevalent than the failure to shift from numbers to letters. Whereas incorrectly sequencing numbers in ascending order hardly ever occurred on* TMT-B* (0% of patients committed ≥ 2 errors), the failure to connect letters in correct alphabetical order happened slightly more often (6.7% of patients committed ≥ 2 errors). Note that these data are merely descriptive and that they are not backed up by statistical analyses.

### 3.2. Lesion Analysis

**Figure 1:**
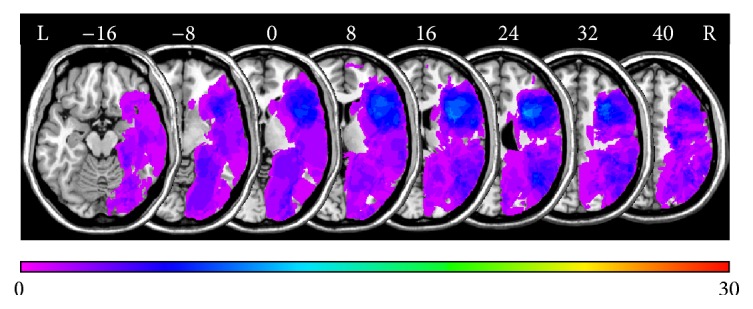
Overlay lesion plots of all thirty patients in* MNI* space. Eight axial slices. The number of overlapping lesions is illustrated by color, from violet (*N* = 1) to red (*N* = 30). Maximum overlap occurred in the right frontal lobe. The area colored light blue indicates overlapping lesions in eleven patients (37% lesion overlap). Numbers indicate MINI coordinates.

Figure 1 shows an overlay lesion plot of all thirty patients in eight axial slices of a standard brain in MNI space. The maximum lesion overlap occurred in the right prefrontal cortex (PFC) where up to eleven patients showed overlapping lesions in single voxels.

**Figure 2:**
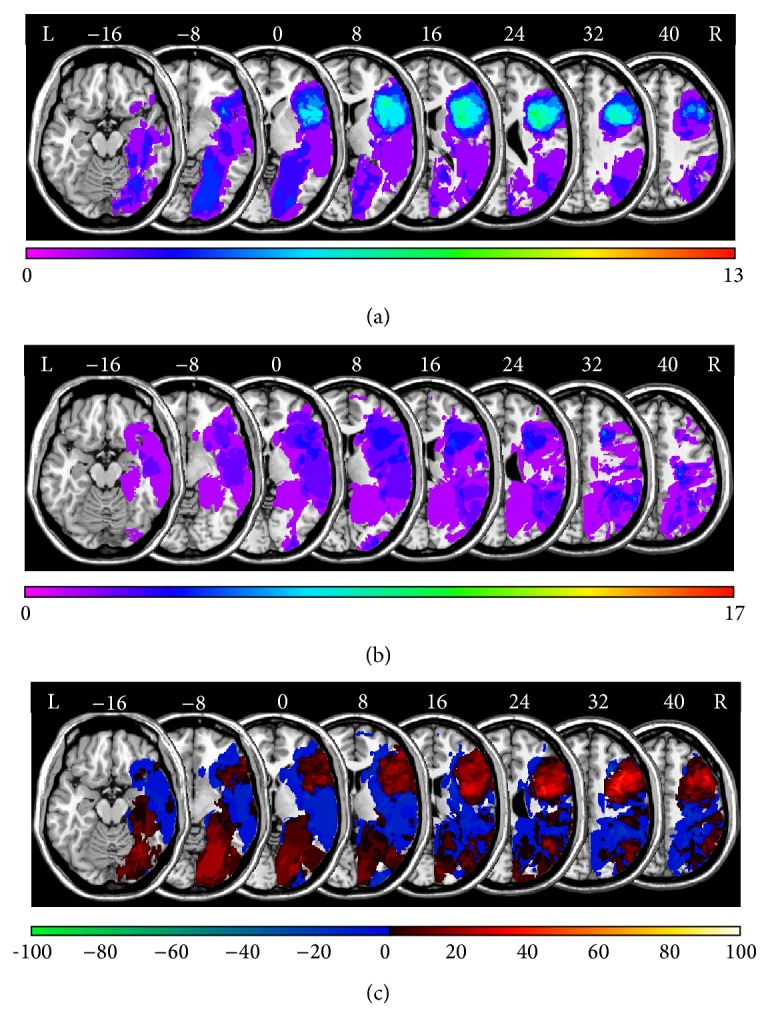
Anatomical results obtained from the lesion subtraction analysis on the number of* TMT-B* total errors. (a) Overlay lesion plots for those patients who committed two or more total errors (*Mdn* = 1) on the* TMT-B* (*N* = 13). The number of overlapping lesions is illustrated by color, from violet (*N* = 1) to red (*N* = 13). (b) Overlay lesion plots for those patients who committed less than two total errors on the* TMT-B* (*N* = 17). The number of overlapping lesions is illustrated by color, from violet (*N* = 1) to red (*N* = 17). (c) Overlay plots of the subtracted superimposed lesions of the patients with two or more total errors on the* TMT-B* minus the patients with less than two total errors on the* TMT-B*. Colors code increasing frequencies from dark-red (difference from 1% to 20%) to white-yellow (difference from 81% to 100%), indicating regions damaged more frequently in patients who committed relatively many total errors on the* TMT-B*. The colors from dark-blue (difference from −1 to −20%) to light-green (difference from −81 to −100%) indicate regions damaged more frequently in patients who committed relatively few total errors on the* TMT-B*.

Figure 2 demonstrates a lesion subtraction analysis for* TMT-B* total errors.  Figure 2(a) shows the overlay lesion plot of patients committing two or more errors on the* TMT-B*, and the overlay lesion plot of patients committing less than two errors is presented in Figure 2(b). The results of subtracting the group with less than two errors from the group with two or more errors are shown in Figure 2(c). The right frontal lobe was more frequently damaged in patients committing two or more errors on the* TMT-B*.

**Figure 3:**
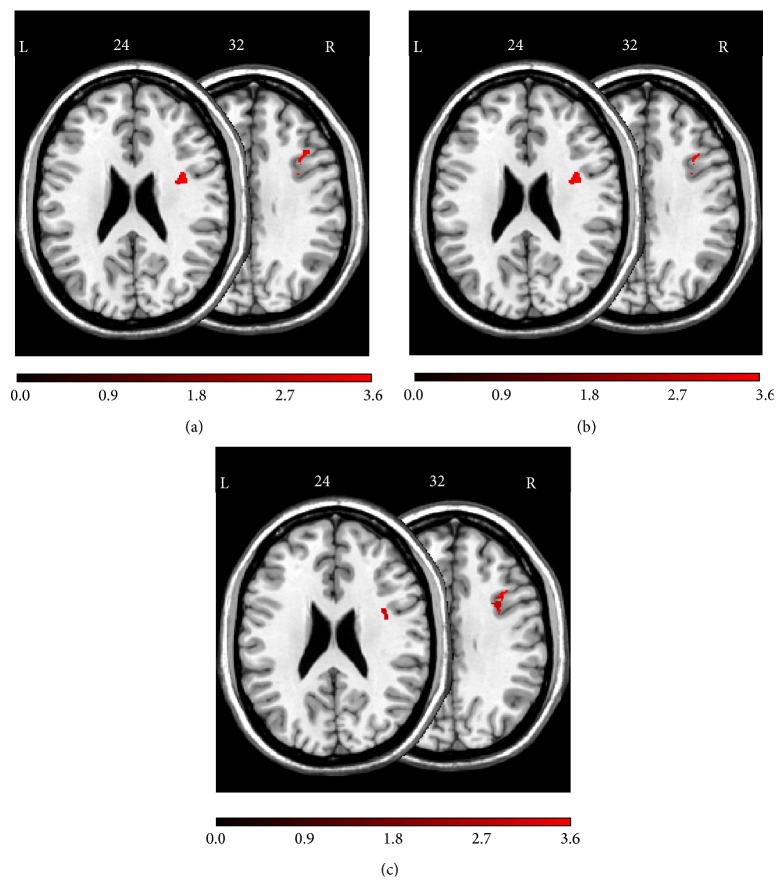
Anatomical results obtained from the voxel-based lesion-behavior mapping (a) on the number of* TMT-B* total errors, (b) on the number of* TMT-B* shifting errors (error type A + type B), and (c) on the number of* TMT-B* sequencing errors (error type C + type D). The location of voxels for which the voxel-based lesion-behavior mapping indicated that the observed* Bz* surpassed *z*_crit_ is shown. See text for details. Numbers indicate MNI coordinates.

A voxel-based lesion-behavior analysis revealed a significant association between voxel damage in the right hemispheric DLPFC and* TMT-B* total errors (cf.  Table 2). The significant voxels' locations are shown in Figure 3(a). The analysis revealed three regions: First, an area around MNI coordinates *X* = 38, *Y* = 2, and *Z* = 24 in the frontal subgyral white matter underneath cortical area BA6. Second, an area around MNI coordinates *X* = 34, *Y* = 5, and *Z* = 32 in the frontal subgyral white matter underneath cortical area BA9. Third, an area around MNI coordinates *X* = 37, *Y* = 17, and *Z* = 32 within the right middle frontal gyrus (cortical area BA9).

Figure 3(b) depicts the voxels' location for which a voxel-based lesion-behavior analysis revealed a significant association between voxel damage and number of* TMT-B *shifting errors (cf.  Table 2). The analysis revealed three regions: First, an area around MNI coordinates *X* = 37, *Y* = 5, and *Z* = 24 in the frontal subgyral white matter underneath cortical area BA6. Second, an area around MNI coordinates *X* = 35, *Y* = 6, and *Z* = 32 within the right inferior frontal gyrus underneath cortical area BA9. Third, an area around MNI coordinates *X* = 37, *Y* = 17, and *Z* = 32 within the right middle frontal gyrus (cortical area BA9).

Figure 3(c) depicts the voxels' location for which the voxel-based lesion-behavior analysis revealed a significant association between voxel damage and number of* TMT-B* sequencing errors (cf.  Table 2). The analysis revealed three regions: First, an area around MNI coordinates *X* = 38, *Y* = 3, and *Z* = 24 in the frontal subgyral white matter underneath cortical area BA6. Second, an area around MNI coordinates *X* = 34, *Y* = 5, and *Z* = 32 in the frontal subgyral white matter underneath cortical area BA9. Third, an area around MNI coordinates *X* = 37, *Y* = 17, and *Z* = 32 within the right middle frontal gyrus (cortical area BA9).

No significant association between voxel damage and the total amount of* TMT-A* errors was found (cf.  Table 2).

Voxel-based lesion-behavior analyses revealed no significant association between voxel damage and* TMT-A* or* TMT-B* completion time (cf.  Table 3).

## 4. Discussion

Our results are congruent with Stuss et al.'s [24], according to which* TMT-B* errors, but not speed measures, are sensitive to DLPFC damage. There are, however, notable differences between these two studies. First, Stuss et al. [24] relied on group analysis treating patients with damage in different brain parts as separate groups. In contrast, voxel-based lesion-behavior mapping analyses whether lesions in individual brain voxels are reliable predictors of behavioral impairments, without any a priori assumptions [26–28]. This improves the anatomical precision for the analysis of lesion location. Second, the patient groups in Stuss et al.'s [24] study were quite heterogeneous with regard to lesion etiology and chronicity, two factors which possibly interact in unknown ways with the number of* TMT-B* errors. In our study, the patient sample was relatively homogeneous, since solely acute stroke patients participated. Further empirical efforts are required to clarify whether the current findings generalize to subacute and chronic stroke patients with prefrontal lesions and to frontal lobe damage of other etiologies. Third, only patients with right hemispheric lesions were included in the current study, whereas patients with lesion in the left or right hemisphere as well as with bilateral lesions participated in Stuss et al.'s [24] study. As the number of* TMT-B* errors was not affected by lesion laterality in this earlier study, the authors concluded unilateral prefrontal lesions of either cerebral hemisphere being associated with impaired* TMT-B* accuracy.

However, this conclusion should be treated cautiously given the available task switching studies. Generally, in task switching experiments, response times (RTs) are slower and response accuracy is often lower for trials requiring task switching compared to task repetition (see [39], for review). Neuropsychological studies of task switching are scarce and their results are contradictory (see [40], for review), possibly due to usually small neurological patient samples. Aron et al. [41] applied a predictable task switching paradigm without explicit task cues (i.e., an endogenous paradigm). Patients with left in contrast to right lateral prefrontal lesions showed significantly larger RT switch costs, whereas the right compared to the left lateral prefrontal group showed dramatically elevated shifting errors on switch trials. Shallice et al. [42] used a task switching paradigm with explicit task cues (i.e., an exogenous paradigm). The authors reported a left lateral prefrontal effect, but this time on errors, whereas the major RT effect was a striking slowing on switch and repeat trials for patients with superior medial prefrontal lesions.

The* TMT-B* should be considered to be an endogenous task switching paradigm. Given our, Stuss et al.'s [24], and Aron et al.'s [41] results, it has been more consistently demonstrated that lateral prefrontal lesions in the right cerebral hemisphere are associated with elevated error rates in endogenous task switching paradigms. However, this lateralization hypothesis (right/left hemispheric lesions are associated with elevated error rates in endogenous/exogenous task switching paradigms) calls for further systematic inquiry.

Our results show that* TMT-B* errors, but not completion times, are associated with DLPFC lesions. Most patients who were included in our study had prefrontal lesions, because we were mainly interested in precisely describing prefrontal areas involved in endogenous set shifting. Our study was not specifically designed to detect reliable brain-behavior-relationships of other, for example, posterior, brain regions. Thus, we do not claim that the documented association between* TMT-B* performance accuracy and prefrontal lesions is* exclusively *related to this particular lesion location; rather, the specificity of the prefrontal brain-behavior-relationship has still to be studied. Nevertheless, the relationship between right frontal damage and errors on the* TMT-B* is of importance against the background that there are few measures available for assessing functional disability in right frontal patients [10, 43, 44]. For example, while verbal fluency can be considered a test of left frontal function [45, 46], nonverbal analogs of verbal fluency, such as design fluency, do not seem to provide comparably sensitive and specific indices of right frontal function [47].

We conclude that acute stroke lesions in the right hemispheric DLPFC (i.e., two regions within the right inferior frontal gyrus (BA9 and BA6)) are associated with enhanced* TMT-B* error proneness (i.e., increased* TMT-B* total and sequencing errors). Furthermore, a region within the right middle frontal gyrus (BA9) predicted the occurrence of* TMT-B* shifting errors. These anatomical data suggest caudal areas within BA9 and rostral areas within BA6 being frontal regions associated with shifting and sequencing errors on the* TMT-B*. In accordance with Stuss et al. [24], the observation of multiple* TMT-B* errors constitutes a behavioral corollary of right hemisphere DLPFC dysfunction. This is remarkable if one considers that there are not many neuropsychological measures possessing documented sensitivity to right hemispheric DLPFC dysfunction [18, 44].

Most of the regions found to be significant from voxel-based lesion-behavior analyses were white matter regions. The importance of white matter disconnection as a pathogenetic mechanism for specific neurological syndromes has been appreciated since long times (e.g., [48]). Our results indicate that white matter disconnection is important for poststroke behavioral deficits. The disconnection of white matter leads to remote physiological effects in regions of cortex that are structurally intact. The cortical distribution of these secondary cortical abnormalities, including the DLPFC, may partly account for the observed behavioral correlations. It is well-established that the DLPFC is implicated as a key node in a frontoparietal network mediating top-down executive control of attention [49]. Damage to the DLPFC, or of white matter beneath the DLPFC, might contribute to the observed behavioral correlations in a variety of ways, as discussed below.

The empirical finding that associates right hemispheric DLPFC lesions with* TMT-B* errors needs explanation by a cognitive theory. First, a major component of the* TMT-B* is shifting between two cognitive sets (numbers and letters). Any failure of cognitive set shifting should lead to a specific accumulation of shifting errors. Second, the* TMT-B* requires serial organization of behavior, under conditions of divided attention and high working memory load. Impaired serial organization of behavior should lead to an accumulation of sequencing errors. However, we observed both, more shifting and more sequencing errors in patients with right hemispheric DLPFC lesions, suggesting that neither a failure of cognitive flexibility nor a failure of serial organization of behavior accounts for our findings. Alternatively, enhanced* TMT-B* error proneness might result from a more general tendency towards stimulus-bound behavior [10]. From this perspective, both error types reflect “false alarms” to distractors via impaired concentration, enhanced distractibility, and/or disturbed response inhibition, that is, neuropsychological dysfunctions, which are often asserted to right hemispheric DLPFC lesions [50, 51].

## 5. Limitations and Conclusions

The data presented here need to be interpreted with caution mainly for three reasons: First, the majority of the patients in the current sample had right frontal lesions, and an extended sample should include both patients with right and left frontal lobe damage in order to examine whether or not our observation is specific for* right* frontal lesions. Second, the extended sample should also include many more patients with right posterior damage in order to examine whether or not our observation is specific for* frontal* lesions. With regard to this issue it would be interesting to know to what extent the increased* TMT-B* error rate in our patients is explained partly by concurrent posterior cortical lesions causing a visuoperceptual deficit. As noted by one of our reviewers, the area of overlapping occipital damage amongst patients was larger and more diffuse than that in prefrontal areas. Thus, our results were obtained in an analysis that was weighted towards highlighting the more concentrated (prefrontal) area. However, damage to multiple sites across the occipital cortex could lead to the same (or similar) visuoperceptual deficit, yet not be highlighted by the analysis used as the lesion sites are more distributed spatially amongst patients. Third, exclusion of patients with visual field defects or with hemispatial neglect generally reduces the generalizability of our claims.

Taken together, our findings suggest that aspects of* TMT* performance, namely, the accuracy on* TMT-B*, are associated with right frontal lobe damage. However, all our patients suffered from damage to the right frontal lobe and we can thus not compare the performance of patients with damage to the right frontal lobe to the performance of patients with damage elsewhere. We can, as a consequence, not draw firm conclusions concerning the specificity of the relationship between damage to the right frontal lobe and the number of errors on* TMT-B*. Specifically, future work should examine* TMT-B* accuracy in patients with left frontal lesions and in patients with posterior lesions.

On a pragmatic basis, our data imply that one should be cautious about concluding the location of a lesion based on completion times in trail making tasks. However,* TMT* ranks fourth among the top ten executive functioning instruments [1], and completion times, but not error scores, received standardization [3]. The subtraction method (*TMT-B* completion time minus* TMT-A* completion time) is widely used in clinical practice; however, the subtraction method should not be pursued for psychometric reasons [9]. The current data suggest that multiple* TMT-B* errors might be a more sensitive indicator of DLPFC dysfunction, as originally conjectured by Stuss and Levine [18]. The finding that right hemispheric DLPFC lesions were associated with enhanced shifting and sequencing errors alike suggests that rather nonspecific attentional impairments underlie enhanced error proneness, as discussed above.

## Figures and Tables

**Table 1:** Demographic and neuropsychological patient characteristics.

	*N*	M	SD
Age	30	60.17	9.70
Sex	30	17 (m)/13 (f)	/
Years of education	30	12.17	2.16
Handedness	30	0.93	0.27
CES-D (*z*)	26	0.10	0.86
MMSE (rs)	30	27.50	2.18
WST (*z*)	28	−0.36	0.83
RWT—subtest s-words (*z*)	30	−0.38	0.65
RWT—subtest animals (*z*)	30	−0.29	0.51
MCST—*N* categories (rs)	26	5.42	1.21
MCST—*N* perseveration errors (rs)	26	1.81	2.33

Center for Epidemiologic Studies Depression Scale [30]; handedness: handedness ratio on the Edinburgh Handedness Questionnaire [31]; −1 = strongly left-handed, 0 = ambidextrous, and 1 = strongly right-handed; Mini-Mental-State-Examination [32]; Modified Card Sorting Test [33]; Regensburger Wortflüssigkeits-Test (Regensburger Word Fluency Test) [34]; Wortschatz-Test (Vocabulary Test) [35].

*Note*. Sex: m = male and f = female; years of education: school and vocational education; *N* = number of subjects; M = Mean; SD = standard deviation; *z* = standardized *z*-score; rs = raw score.

**Table 2:** Neuropsychological results (number of subjects) and Brunner-Munzel test statistics (max. *z*, *z*_crit_) over various *TMT* error scores.

*TMT* error scores	no errors	1 error	≥2 errors	max. z	z_crit_
*TMT-A*, sequencing errors (type C)	26	2	2	2.425	3.452
*TMT-B*, total errors	9	8	13^+^	3.972^∗^	3.501
*TMT-B*, shifting errors (type A)	18	9	2	3.084	3.381
*TMT-B*, shifting errors (type B)	20	6	4	4.163^∗^	3.285
*TMT-B*, shifting errors (type A + B)	16	7	7	3.233^∗^	2.996
*TMT-B*, sequencing errors (type C)	28	2	0	3.154	3.154
*TMT-B*, sequencing errors (type D)	22	6	2	4.250^∗^	3.403
*TMT-B*, sequencing errors (type C + D)	20	8	2	3.724^∗^	3.285

Note: ^∗^ *p* < .05. ^+^All patients who made two or more than two errors of either type on the *TMT-B* (i.e., *TMT-B* total errors) had frontal lesions in Stuss et al.'s [24] study.

**Table 3:** Neuropsychological results and Brunner-Munzel test statistics (max. *z*, *z*_crit_) over various *TMT* time scores.

*TMT* time scores	M	SD	Mdn	IQR	max. z	z_crit_
*TMT-A*, completion time (sec)	82.95	122.15	48.10	61.02	2.549	3.121
*TMT-B*, completion time (sec)	184.51	153.01	134.13	114.36	3.320	3.320

Note: IQR = interquartile range (Q_75_–Q_25_).

## Endnotes

1. A possible statistical solution to the problem would be to use the severity of hemiparesis, apraxia, aphasia, pain, hemianopia, neglect, and other neuropsychological disturbances as covariates. However, covariance analysis presupposes the separation of patients into meaningful groups of individuals, as in neuropsychological group studies, and it further requires a number of restrictive conditions to be met, for example, that the slopes of the regression lines (which relate covariates and dependent variables) fitted to the groups, to be parallel.

## Abbreviations

BA: Brodmann's area
BM: Brunner-Munzel test
CES-D: Center for Epidemiologic Studies Depression Scale
CT: Computed tomography
DLPFC: Dorsolateral prefrontal cortex
DWI: Diffusion-weighted imaging
EPI: Echo planar imaging
FLAIR: Fluid-attenuated inversion-recovery imaging
FOV: Field of view
MCST: Modified Card Sorting Test
MMSE: Mini-Mental-State-Examination
MNI: Montreal Neurological Institute
MRI: Magnetic resonance imaging
PFC: Prefrontal cortex
RT: Response time
RWT: Regensburger Wortflüssigkeits-Test (Regensburger Word Fluency Test)
TE: Echo times
TR: Repetition time
*TMT*: Trail Making Test
WCST: Wisconsin Card Sorting Test
WST: Wortschatz-Test (Vocabulary Test).
